# Trends in Hospital Admissions for Chronic Obstructive Pulmonary Disease in Men and Women in Spain, 1998 to 2018

**DOI:** 10.3390/jcm10071529

**Published:** 2021-04-06

**Authors:** Domingo Orozco-Beltrán, Juan Manuel Arriero-Marin, Concepción Carratalá-Munuera, Juan J. Soler-Cataluña, Adriana Lopez-Pineda, Vicente F. Gil-Guillén, Jose A. Quesada

**Affiliations:** 1 Department of Clinical Medicine, Miguel Hernández University, 03550 San Juan de Alicante, Spain; dorozcobeltran@gmail.com (D.O.-B.); arriero_jma@gva.es (J.M.A.-M.); adriannalp@hotmail.com (A.L.-P.); vte.gil@gmail.com (V.F.G.-G.); jquesada@umh.es (J.A.Q.); 2 Pneumology Department, University Hospital of San Juan de Alicante, 03550 San Juan de Alicante, Spain; 3 Pneumology Department, Arnau de Vilanova-Lliria Hospital, 46160 Valencia, Spain; jjsoler@telefonica.net

**Keywords:** pulmonary disease, chronic obstructive, inpatients, sex, disease progression

## Abstract

The prevalence of chronic obstructive pulmonary disease (COPD) is rising faster in women in some countries. An observational time trends study was performed to assess the evolution of hospital admissions for COPD in men and women in Spain from 1998 to 2018. ICD-9 diagnostic codes (490–492, 496) from the minimum basic data set of hospital discharges were used. Age-standardised admission rates were calculated using the European Standard Population. Joinpoint regression models were fitted to estimate the annual percent change (APC). In 2018, the age-standardised admission rate per 100,000 population/year for COPD was five times higher in men (384.8, 95% CI: 381.7, 387.9) than in women (78.6, 95% CI: 77.4, 79.9). The average annual percent change (AAPC) was negative over the whole study period in men (−1.7%/year, 95% CI: −3.1, −0.2) but positive from 2010 to 2018 (1.1%/year, 95% CI: −0.8, 2.9). In women, the APC was −6.0% (95%CI: −7.1, −4.9) from 1998 to 2010, but the trend reversed direction in the 2010–2018 period (7.8%/year, 95% CI: 5.5, 10.2). Thus, admission rates for COPD decreased from 1998 to 2010 in both men and women but started rising again until 2018, modestly in men and sharply in women.

## 1. Introduction

The Chronic obstructive pulmonary disease (COPD) is one of the main causes of morbimortality worldwide, ranking fourth (and likely third in coming years) among the leading causes of death globally [1] and affecting over 380 million people [2]. COPD exacerbations are associated with impaired lung function [2] and quality of life [3], increased hospitalisations [4], and mortality [5], and significant health system costs [6].

In one study of more than 53 million patients of 8064 GPs in the UK in 2006 to 2009, the admission rate for COPD per 100,000 population/year was 265, ranging from 124.7 to 646.5, depending on the centre, and from 0 to 2175.2 according to the physician [7]. There was a positive association between admissions and the proportion of patients with no previous diagnosis of COPD, smoking, and low socioeconomic status, while protective factors included the flu vaccine, certain GP characteristics, and the accessibility of the consult. However, the influence of gender was not analysed. Another study, also based in the NHS, found that one-year mortality after an admission due to a COPD exacerbation was 24.1%, and it was associated with more advanced age, male sex, comorbidities, greater length of stay, and having missed previous appointments [8]. A previous study in 7002 patients attended in 72 primary health care centres in Scotland from 2000 to 2008 and followed to 2010, reported that 25% of patients were admitted for a COPD exacerbation, with no differences in risk between gender [9]. In another study, this time in Canada, 60.1% of patients admitted for COPD were men, but there were no gender-related differences in the rate of readmissions [10]. In a Spanish hospital, Abadías Medrano et al. [11] followed 216 patients who presented to the emergency department (ED) for a COPD exacerbation in May and June 2016; 84% were men, although there was no difference according to gender in the proportion who were admitted. Compared to men, women were less likely to be smokers (9% vs. 63%), more likely to present with the asthma/COPD phenotype (48% vs. 11%), and earlier to see their doctor following discharge (4 days versus 7 days), with no gender differences with regard to history of COPD exacerbations over the previous year. Men were at greater risk for readmission at 90 days (8.2% versus 4.7%) [11]. A recent systematic review of COPD [12] exacerbations in international clinical trials, which included 55 studies, found a 6.7% (95% CI 4.4, 9.0; *p* < 0.001) annual decrease in admissions from 1997 to 2017, equivalent to a 50% (95% CI 36%, 61%) decrease over a decade. This review had some limitations, including a small number of studies, different for each year; the lack of analysis by gender; and the failure to include gender in multivariable analyses, which adjusted only for age, smoking, symptoms, and lung function [12].

Although COPD affects many more men than women, a recent review of the influence of female sex on respiratory diseases [13] noted that prevalence of COPD is rising faster in women, especially young women. In another study from the USA [14], authors found that the rates of hospital admissions and mortality due to COPD in women were actually exceeding those seen in men. Moreover, the group of never-smokers with COPD was predominated by women [15].

The aim of this study is to assess the evolution of hospital admissions due to COPD according to gender from 1998 to 2018 and to precisely describe the time trends of this problem over the 21-year study period.

## 2. Materials and Methods

Observational time trends study of hospital admissions due to COPD from 1998 to 2018. Data were drawn from the minimum basic data set of hospital discharges [16]. The diagnostic codes used were from the International Classification of Diseases, 9th revision (ICD-9 490: bronchitis, not specified as acute or chronic; 491: chronic bronchitis; 492: emphysema; and 496: chronic airway obstruction, not elsewhere classified). In Spain, the minimum basic data set has been mandatory since 1992. The variables collected were: gender (man/woman), age (years), year of admission (1998 to 2018), and cause of admission (ICD-9: 490–492, 496). The population for each year was determined using statistical data from the continuous census register, managed by the National Statistics Institute. Inclusion criteria were age of 45 years or more and emergency admission from 1998 to 2018. People with missing data or unknown values for any of the study variables were excluded.

### Statistical Analysis

A descriptive analysis was undertaken, calculating frequencies for qualitative variables and minimum, maximum, mean and standard deviation (SD) values for quantitative variables. Direct age-standardised admission rates (95% confidence intervals [CIs]) were determined using the 2013 European Standard Population for the age groups 45–64 years, 65–84 years, and ≥85 years, and results for each study year were stratified by gender.

SPSS v.25 statistical software was used to calculate the admission rates. To analyse the time trends for hospital admissions during the study period and detect significant changes in their direction, we fitted joinpoint regression models for both the total study population and by age group and gender. These models estimate the annual percentage of change (APC) and 95% CIs for the age-standardised admission rates in each segment detected. A negative APC indicates a downward trend, and a positive value, an upward trend. The models also show the average annual percent change (AAPC) for the overall study period. Models were fitted with the assumption of uncorrelated errors, and they were selected using the permutation test, with a minimum number of 0 joinpoints and a maximum of 3. For this step, we used the US National Cancer Institute’s Joinpoint Regression Program v.4.6.0 [17].

## 3. Results

Table 1 shows the trend in hospital admissions for COPD over the study period. In men, the joinpoint regression shows a significant negative APC, of −1.7%. However, from 2010 the negative trend is inverted by a slightly positive trend, accentuated in men over 85 years of age. In women, the 1998–2010 period is also characterised by decreasing admissions (APC −6.0%), but as with men, the following years see a statistically significant reversal, with an APC of 7.8%. The highest increase is in the 45–64-year age group.

Table 2 and Table 3 describe hospital admissions for COPD from 1998 to 2018 along with the age-standardised rates in men (Table 2) and women (Table 3). Data are presented for the overall sample and stratified by gender. In 1998, there were a total of 66,714 hospital admissions due to COPD in Spain (54,237 in men and 12,477 in women), while in 2018, there were 77,134 (60,944 in men and 16,190 in women). Age-standardised admission rates per 100,000 population/year were five times higher in men (384.8, 95% CI 381.7, 387.9) than in women (78.6, 95% CI 77.4, 79.9) for that year. These admission rates represent a net decrease from 1998 (men: 505.9, 95% CI 501.6, 510.3; women: 83.6, 95% CI 82.1, 85.0); however, the 2018 rates are higher than those from 2010 (men: 346.5, 95% CI 343.4, 349.7; women: 43.9, 95% CI 42.9, 44.8).

Table 4 shows the distribution by age groups; the admissions in younger women (45–64 years) tripled over the study period, while in men, admissions rose fastest in the oldest age group, also by three-fold.

**Figure 1:**
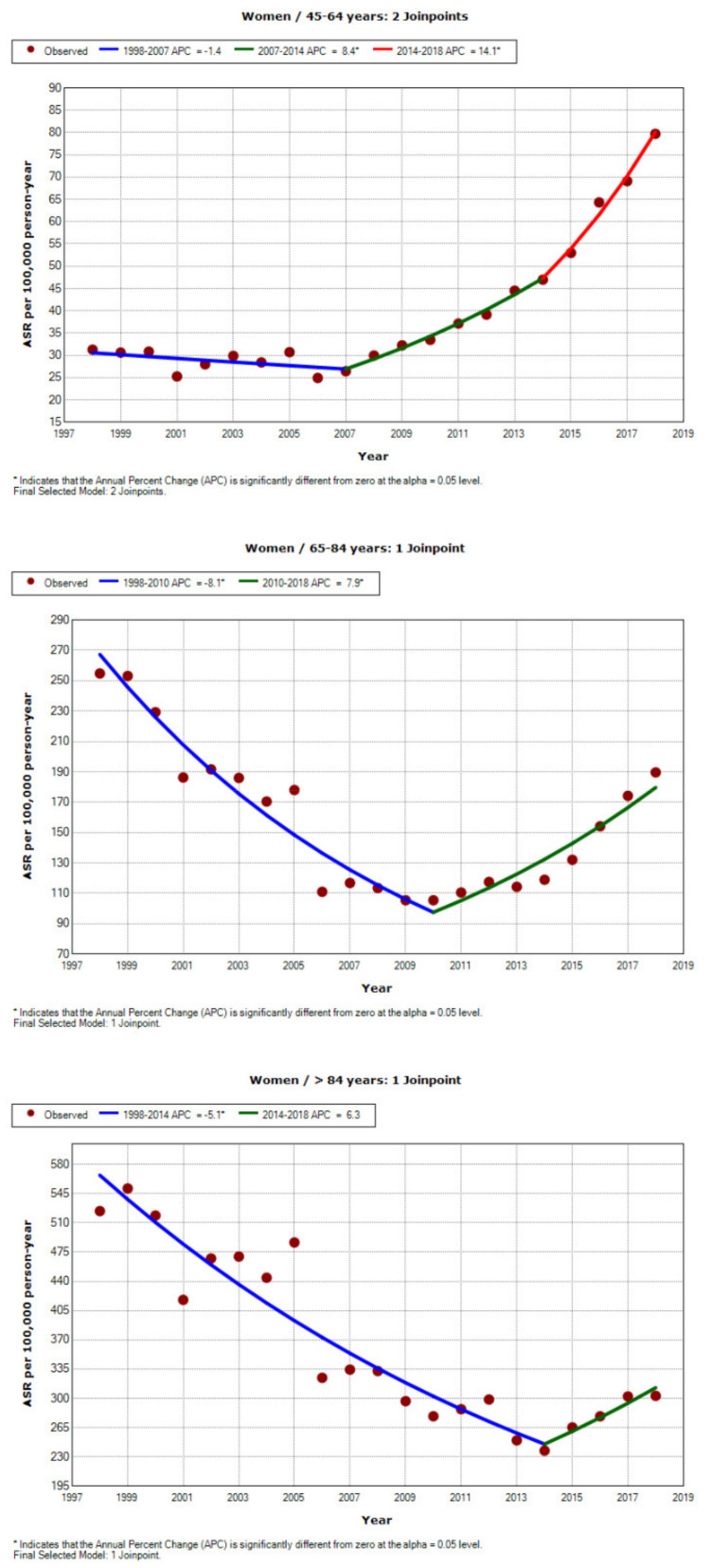
Joinpoint regression analysis of trends in hospital admission due to COPD in women Spain from 1998 to 2018, by age group.

**Figure 2:**
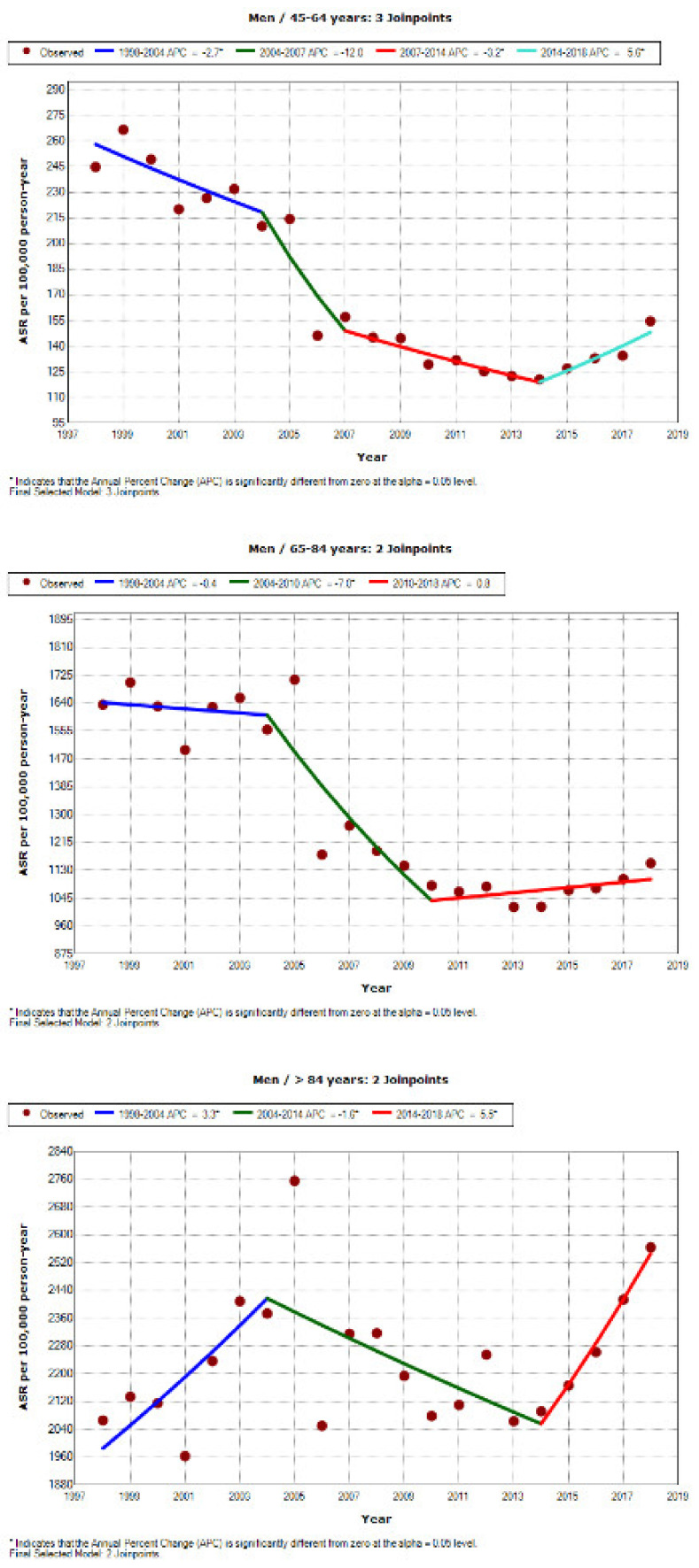
Joinpoint regression analysis of trends in hospital admission due to COPD in men Spain from 1998 to 2018, by age group.

Figure 1 and Figure 2 show the APCs over the study period graphically and by age group and gender. In women (Figure 1), there is a clear decrease in admissions from 1998 to 2006. After that, the trend is uneven in different age brackets until around 2008, when rates begin to trend upward, affecting all groups. The increase is especially notable in women aged 45 to 64 years, that is, in the youngest age group studied; the increase in admissions is significant and continuous from 2007 until study end. In men (Figure 2), the decline in admissions among the youngest age group (45–64 years) is evident from 1998 to 2014, after which admission rates begin a gentle rise until 2018. In men aged 65–84 years, the decrease in admissions also starts to stabilise around 2010, and in the oldest men, substantial variability until 2013 gives way to a clear upward trend in admissions.

## 4. Discussion

From 1998, the overall rate of hospital admissions due to COPD in Spain declined until around 2010, when the trend reversed, moderately in men and more sharply in women. This observation has not been previously described in Spain. Previous studies in our country have focused on earlier and shorter time periods. In a study published in 2013, Miguel-Diez et al. [18] analysed the period from 2006 to 2010, observing that the incidence of hospital admissions per 10,000 population/year due to COPD exacerbations decreased from 2.9 to 2.4. In men, this reduction was larger (38.7 to 32.4) than in women (5.5 to 4.7), and the mean cost per patient increased from EUR 3147 to EUR 4129. Librero et al. [19] analysed a longer period (2002 to 2013), showing how the decrease in the number of admissions for COPD in women began to slow around 2010. Our study encompasses an even longer period and includes data from more recent years (up to 2018), showing a quite clear increase from around 2009.

The explanation for the increase in COPD admissions in women in recent years is not simple. It could arise from the increase in smoking rates three or four decades ago in women, with the subsequent increase in COPD prevalence. This fact is especially worrying considering that admission rates do not appear to have peaked yet. Moreover, currently adolescent girls have a higher prevalence of smoking than adolescent boys, and the rate of underdiagnosis for COPD is higher in women than in men [20], which could be associated with more admissions. Furthermore, women with COPD present a higher prevalence of anxiety and depression; these disorders have a greater impact on women’s quality of life; and women present more dyspnoea than men with the same lung function impairment [21]. A 2018 meta-analysis showed an association between anxiety and depression, on the one hand, and the incidence of COPD exacerbations, on the other [22]. Women appear to be more susceptible to the consequences of smoking [14]. Moreover, gender-related differences have been detected in the use of health services, with women more likely to receive the wrong diagnosis [23] and perceive diagnostic delays; this was attributed to the lack of available professionals and worse insurance coverage, although the latter is not applicable to the Spanish health system model [24]. Another factor to consider could be the level of vitamin D: in one meta-analysis, review authors observed an inverse association between vitamin D levels and the incidence of COPD exacerbations and a protective role for vitamin D supplements in patients with COPD and vitamin D deficiency [25]. In an earlier study, authors observed low levels of vitamin D in the female population of Spain [26]. Finally, numerous studies across different pathologies have found that women are at greater risk of not adhering to treatment, which could also have an impact on disease control [27]. Although socioeconomic factors could certainly influence adherence, the differences seem to persist even after adjusting for this variable [28].

By age group, there was a three-fold rise in the proportion of COPD admissions among women in the youngest age group (45–64 years), which grew from 11.6% in 1998 to 32.3% in 2018. This result could support the hypothesis that the higher rates in COPD admissions in women is attributable to the increased prevalence of smoking in young women.

Internationally, a growing body of research focuses specifically on the impact of COPD in women, with evidence pointing to rising prevalence, morbidity and mortality since the early 2000s [29]. A study in eight countries (Spain, Italy, France, Germany, the Netherlands, the UK, the USA, and Canada) found that just 40% of participants reported having undergone a spirometry test. Once again, gender biases put women at a disadvantage. Researchers found that even after adjusting for disease severity and smoking, women were less likely than men to have had this test (OR 0.84, 95% CI 0.72, 0.98) [30]. In Spain, one study estimated that 73% of patients with spirometric findings consistent with COPD were not diagnosed with this disease, and this percentage was higher in women (86.0% versus 67.6%; *p* < 0.05) [31]. In the USA, COPD has become the first cause of mortality in women [32]; with the number of COPD deaths quadrupling since 1980 and exceeding those in men every year since 2000. Women develop COPD at an earlier age than men, and women find it harder to quit smoking [33].

In men, there was a clear decrease in the admissions rates for COPD over the whole study period. This drop could be attributed to a fall in prevalence; however, the data indicate that COPD prevalence is increasing. Thus, it appears that improvements in the quality of health care and COPD treatments could be indirect contributors to the number of exacerbations and in turn, hospital admissions. Other factors at play might include the implementation of day hospitals, home care units, the increased involvement of primary health care, chronic disease management programmes (including for COPD), improved flu vaccination rates, and new organisational forms like care pathways and other continuous care programmes. A 2019 meta-analysis of clinical trials [12] that tested interventions for improving COPD management also revealed a decrease in exacerbation rates over the past two decades; suggested factors contributing to this decline were flu vaccinations, physical activity, increased use of short acting bronchodilators, better air quality, and healthier behaviours. Another recent study [34] showed that applying “care bundles” encompassing 10 interventions (5 related to the admission and 5 activities to be performed before discharge) did not manage to reduce readmission rates (odds ratio 1.02, 95% CI 0.83, 1.26) or mortality, and it was not cost-effective. However, it did reduce the rate of emergency department visits (risk ratio 0.63, 95% CI 0.56, 0.71). For their part, Ospina et al. [35] performed a systematic review with 14 studies, analysing interventions based on care bundles implemented upon discharge; review authors concluded that this intervention reduced readmissions but did not improve mortality or quality of life.

The proportion of hospital admissions in the oldest men (≥85 years) tripled over the study period, from 7.8% in 1998 to 20.5% in 2018. This greater representation of very old men could be due to increased longevity; Spain is the EU member state with the longest life expectancy—83.4 years, higher than the EU average of 80.9 (2017) and 4 years longer than it was in 2001 (79.4 years). Moreover, after France, Spain is the country with the longest life expectancy at age 65—21.5 years [36].

All these data suggest the need for gender-specific approaches to COPD. This serious public health problem demands updating the knowledge base and adapting its application to epidemiological changes [37]. Health services must intensify their efforts to systematically apply recommendations from clinical guidelines [38] to women.

### Limitations

One limitation of this kind of study is that it collects diagnoses generated by a diverse range of specialists, and it is not possible to guarantee that the COPD diagnosis was always based on objective tests such as spirometry. However, given that our focus was on hospital admissions, not ambulatory care, it is likely that these tests were in fact undertaken, as inpatient examinations tend to be more complete than outpatient services. Furthermore, the annual trends identified in this study should be completely reliable, as the sample was large enough to minimise the impact of any potential bias and reveal significant trends at a national level.

## 5. Conclusions

Our analysis of age-standardised admission rates for COPD in Spain over a 21-year period (1998 to 2018) showed a clear decline in both men and women until 2009. However, from 2010 the trend changed direction, modestly in men and sharply in women. The greatest increases in admission rates were in very old men (≥85 years) and middle-aged women (45–64 years); the proportion of these age brackets tripled relative to all admissions in their respective genders in 2018.

## Figures and Tables

**Table 1:** Evolution of hospital admissions due to chronic obstructive pulmonary disease (COPD) in Spain, 1998 to 2018. Trend segments and annual percentage of change (APC) obtained by means of joinpoint regressions for age-standardised admission rates, by gender.

	Segment 1			Segment 2			Segment 3			Segment 4			Overall (1998–2018)	
Group	Period	APC	95% CI	Period	APC	95% CI	Period	APC	95% CI	Period	APC	95% CI	AAPC	95% CI
*Women*														
Total	1998–2010	−6.0 *	(−7.1, −4.9)	2010–2018	7.8 *	(5.5, 10.2)							−0.7	(−1.7, 0.3)
45–64 years	1998–2007	−1.4	(−2.9, 0.1)	2007–2014	8.4 *	(5.9, 10.9)	2014–2018	14.1 *	(10.4, 17.9)				4.9 *	(3.8, 6.1)
65–84 years	1998–2010	−8.1 *	(−9.2, −6.9)	2010–2018	7.9 *	(5.3, 10.7)							−2.0 *	(−3.1, −0.8)
≥85 years	1998–2014	−5.1 *	(−6.0, −4.2)	2014–2018	6.3	(−1.7, 14.9)							−2.9 *	(−4.5, −1.4)
*Men*														
Total	1998–2004	−0.3	(−3.1, 2.6)	2004–2010	−6.5 *	(−10.0, −2.8)	2010–2018	1.1	(−0.8, 2.9)				−1.7 *	(−3.1, −0.2)
45–64 years	1998–2004	−2.7 *	(−4.3, −1.2)	2004–2007	−12.0	(−25.4, 3.8)	2007–2014	−3.2 *	(−5.0, −1.3)	2014–2018	5.6 *	(1.7, 9.7)	−2.7 *	(−5.0, −0.4)
65–84 years	1998–2004	−0.4	(−3.1, 2.4)	2004–2010	−7.0 *	(−10.4, −3.5)	2010–2018	0.8	(−1.1, 2.6)				−2.0 *	(−3.4, −0.6)
≥85 years	1998–2004	3.3 *	(0.3, 6.4)	2004–2014	−1.6 *	(−2.8, −0.4)	2014–2018	5.5 *	(1.3, 9.9)				1.3 *	(0.0, 2.5)

CI: confidence interval; AAPC: annual average percent change; * *p* < 0.05.

**Table 2:** Annual hospital admissions due to COPD and age-standardised rates (ASR) per 100,000 women/year in women, 1998 to 2018.

Year	Total			45–64 Years			65–84 Years			≥85 Years		
	n	ASR	95% CI	n	ASR	95% CI	n	ASR	95% CI	n	ASR	95% CI
1998	12,477	83.6	(82.1, 85.0)	1420	31.3	(29.6, 32.9)	8477	254.8	(249.3, 260.2)	2383	524.1	(503.0, 545.1)
1999	12,942	83.7	(82.3, 85.2)	1398	30.6	(29.0, 32.2)	8720	253.1	(247.8, 258.4)	2642	551.2	(530.2, 572.2)
2000	12,241	77.7	(76.4, 79.1)	1390	30.8	(29.2, 32.5)	8033	229.2	(224.2, 234.3)	2612	518.8	(498.9, 538.7)
2001	10,299	63.4	(62.2, 64.6)	1155	25.3	(23.8, 26.7)	6714	186.3	(181.8, 190.7)	2210	417.9	(400.4, 435.3)
2002	11,103	67.1	(65.9, 68.4)	1282	28.0	(26.5, 29.5)	7080	191.6	(187.2, 196.1)	2496	467.4	(449.1, 485.7)
2003	11,240	66.5	(65.3, 67.8)	1403	29.9	(28.3, 31.5)	7051	186.0	(181.6, 190.3)	2555	469.7	(451.5, 487.9)
2004	10,655	61.8	(60.6, 63.0)	1381	28.4	(26.9, 29.9)	6580	170.4	(166.3, 174.6)	2469	444.3	(426.8, 461.9)
2005	11,584	65.7	(64.5, 66.9)	1565	30.7	(29.2, 32.2)	6982	178.1	(173.8, 182.3)	2781	486.6	(468.5, 504.6)
2006	7852	43.8	(42.8, 44.8)	1291	24.9	(23.6, 26.3)	4421	111.0	(107.7, 114.3)	1933	324.6	(310.2, 339.1)
2007	8415	46.1	(45.1, 47.1)	1414	26.4	(25.1, 27.8)	4699	116.9	(113.5, 120.3)	2056	334.4	(319.9, 348.8)
2008	8584	46.2	(45.3, 47.2)	1648	30.0	(28.5, 31.4)	4576	113.5	(110.1, 116.8)	2158	332.7	(318.6, 346.7)
2009	8348	44.2	(43.2, 45.1)	1815	32.3	(30.8, 33.7)	4302	105.5	(102.3, 108.7)	2019	296.6	(283.7, 309.5)
2010	8434	43.9	(42.9, 44.8)	1919	33.5	(32.0, 35.0)	4338	105.5	(102.3, 108.7)	1993	278.5	(266.3, 290.8)
2011	9069	46.4	(45.4, 47.4)	2166	37.1	(35.6, 38.7)	4584	110.6	(107.3, 113.8)	2152	286.9	(274.8, 299.1)
2012	9715	49.0	(48.0, 50.0)	2309	39.1	(37.5, 40.7)	4890	117.5	(114.1, 120.9)	2345	298.6	(286.5, 310.6)
2013	9663	48.6	(47.6, 49.6)	2670	44.5	(42.8, 46.2)	4778	114.3	(111.0, 117.6)	2025	249.7	(238.8, 260.6)
2014	9934	49.9	(48.9, 50.9)	2827	47.0	(45.2, 48.7)	4946	119.0	(115.6, 122.3)	2005	237.4	(227.0, 247.8)
2015	11,219	55.7	(54.6, 56.7)	3240	53.0	(51.2, 54.8)	5501	132.1	(128.5, 135.7)	2331	265.2	(254.4, 275.9)
2016	13,130	65.0	(63.9, 66.2)	4007	64.3	(62.3, 66.3)	6370	154.1	(150.3, 158.0)	2533	278.4	(267.6, 289.3)
2017	14,649	71.5	(70.4, 72.7)	4388	69.1	(67.1, 71.2)	7243	174.2	(170.2, 178.3)	2842	302.3	(291.2, 313.4)
2018	16,190	78.6	(77.4, 79.9)	5165	79.7	(77.5, 81.9)	7868	189.6	(185.4, 193.9)	2947	303.0	(292.0, 313.9)

n: number of admissions; CI: confidence interval.

**Table 3:** Annual hospital admissions due to COPD and age-standardised rates (ASR) per 100,000 men/year in men, 1998 to 2018.

Year	Total			45–64 Years			65–84 Years			>84 Years		
	n	ASR	95% CI	n	ASR	95% CI	n	ASR	95% CI	n	ASR	95% CI
1998	54,237	505.9	(501.6, 510.3)	10,441	244.8	(240.1, 249.5)	39,043	1635.0	(1618.6, 1651.4)	4186	2065.9	(2003.3, 2128.5)
1999	58,865	530.4	(526.0, 534.8)	11,382	266.5	(261.6, 271.5)	42,322	1702.8	(1686.4, 1719.2)	4541	2133.8	(2071.7, 2195.8)
2000	57,118	507.9	(503.6, 512.1)	10,510	249.2	(244.4, 254.0)	41,321	1629.7	(1613.9, 1645.6)	4674	2114.9	(2054.3, 2175.6)
2001	53,765	464.6	(460.6, 468.7)	9453	220.0	(215.6, 224.5)	39,122	1497.2	(1482.2, 1512.1)	4532	1962.9	(1905.7, 2020.0)
2002	59,332	503.6	(499.5, 507.8)	9766	226.6	(222.1, 231.1)	43,746	1628.0	(1612.7, 1643.4)	5180	2236.2	(2175.3, 2297.1)
2003	62,286	517.2	(513.0, 521.3)	10,245	231.9	(227.4, 236.4)	45,666	1655.7	(1640.5, 1671.0)	5655	2408.2	(2345.4, 2471.0)
2004	59,561	487.2	(483.2, 491.2)	9618	210.1	(205.9, 214.3)	43,694	1558.9	(1544.3, 1573.5)	5632	2373.2	(2311.2, 2435.2)
2005	66,537	533.7	(529.5, 537.8)	10,350	214.3	(210.2, 218.4)	48,840	1711.3	(1696.1, 1726.5)	6727	2754.8	(2689.0, 2820.7)
2006	47,331	371.9	(368.5, 375.3)	7159	146.1	(142.7, 149.5)	34,456	1178.0	(1165.5, 1190.4)	5252	2050.2	(1994.7, 2105.6)
2007	52,204	403.1	(399.6, 406.6)	7937	157.0	(153.6, 160.5)	37,537	1266.1	(1253.2, 1278.9)	6263	2314.7	(2257.4, 2372.1)
2008	50,579	382.4	(379.0, 385.8)	7580	145.1	(141.8, 148.3)	35,840	1189.3	(1177.0, 1201.7)	6703	2316.7	(2261.2, 2372.1)
2009	49,994	368.8	(365.6, 372.1)	7717	144.6	(141.3, 147.8)	34,982	1144.2	(1132.2, 1156.2)	6760	2193.4	(2141.1, 2245.7)
2010	47,808	346.5	(343.4, 349.7)	7026	129.3	(126.2, 132.3)	33,502	1083.5	(1071.8, 1095.1)	6819	2078.2	(2028.9, 2127.5)
2011	48,722	344.3	(341.2, 347.4)	7239	131.8	(128.7, 134.8)	33,694	1064.9	(1053.5, 1076.3)	7337	2110.0	(2061.7, 2158.3)
2012	50,298	349.7	(346.6, 352.8)	6996	125.3	(122.3, 128.2)	34,616	1080.1	(1068.7, 1091.6)	8311	2254.4	(2206.0, 2302.9)
2013	48,269	329.3	(326.3, 332.2)	6955	122.5	(119.6, 125.4)	33,007	1018.2	(1007.2, 1029.3)	7934	2063.2	(2017.8, 2108.7)
2014	48,723	329.6	(326.6, 332.5)	6862	120.5	(117.6, 123.4)	33,094	1018.9	(1007.8, 1029.9)	8415	2091.6	(2046.9, 2136.2)
2015	51,740	344.9	(341.9, 347.9)	7320	126.9	(124.0, 129.9)	34,917	1069.0	(1057.7, 1080.3)	9179	2166.5	(2122.1, 2210.8)
2016	53,740	351.3	(348.3, 354.3)	7859	132.9	(130.0, 135.9)	35,522	1075.0	(1063.7, 1086.2)	10,027	2262.1	(2217.8, 2306.3)
2017	56,347	362.8	(359.8, 365.8)	8123	134.4	(131.4, 137.3)	36,684	1103.4	(1092.0, 1114.8)	11,194	2413.0	(2368.3, 2457.7)
2018	60,944	384.8	(381.7, 387.9)	9551	154.6	(151.5, 157.7)	38,623	1151.9	(1140.3, 1163.4)	12,451	2563.7	(2518.7, 2608.8)

n: number of admissions; CI: confidence interval.

**Table 4:** Evolution of hospital admissions due to COPD in Spain, by gender and age group.

	45–64 Years N (row %)	65–84 Years N (row %)	>84 Years N (row %)	Total N
*Women* 1998	1420 (11.6)	8477 (69.0)	2383 (19.4)	12,280
2010	1919 (23.3)	4338 (52.6)	1993 (24.2)	8250
2018	5165 (32.3)	7868 (49.2)	2947 (18.4)	15,980
*Men* 1998	10,441 (19.5)	39,043 (72.7)	4186 (7.8)	53,670
2010	7026 (14.8)	33,502 (70.8)	6819 (14.4)	47,347
2018	9551 (15.8)	38,623 (63.7)	12,451 (20.5)	60,625

## Data Availability

Publicly available datasets were analyzed in this study. This data can be found here: https://pestadistico.inteligenciadegestion.mscbs.es/publicoSNS/Comun/ArbolNodos.aspx?idNodo=6383 [Last accessed on 2021 Feb 7].
